# Conditioned media from human palatine tonsil mesenchymal stem cells regulates the interaction between myotubes and fibroblasts by IL‐1Ra activity

**DOI:** 10.1111/jcmm.12947

**Published:** 2016-09-13

**Authors:** Kyung‐Ah Cho, Minhwa Park, Yu‐Hee Kim, So‐Youn Woo, Kyung‐Ha Ryu

**Affiliations:** ^1^ Department of MicrobiologySchool of MedicineEwha Womans UniversitySeoulKorea; ^2^ Department of PediatricsSchool of MedicineEwha Womans UniversitySeoulKorea

**Keywords:** human palatine tonsil‐derived mesenchymal stem cells, fatty acid, skeletal muscle, fibroblast, IL‐1β, IL‐1Ra

## Abstract

Saturated free fatty acids (FFAs) act as lipid mediators and induce insulin resistance in skeletal muscle. Specifically, in obesity‐related diseases such as type 2 diabetes, FFAs directly reduce insulin sensitivity and glucose uptake in skeletal muscle. However, the knowledge of how FFAs mediate inflammation and subsequent tissue disorders, including fibrosis in skeletal muscle, is limited. FFAs are a natural ligand for toll‐like receptor 2 (TLR2) and TLR4, and induce chronic low‐grade inflammation that directly stimulates skeletal muscle tissue. However, persistent inflammatory stimulation in tissues could induce pro‐fibrogenic processes that ultimately lead to perturbation of the tissue architecture and dysfunction. Therefore, blocking the link between inflammatory primed skeletal muscle tissue and connective tissue might be an efficient therapeutic option for treating obesity‐induced muscle inactivity. In this study, we investigated the impact of conditioned medium obtained from human palatine tonsil‐derived mesenchymal stem cells (T‐MSCs) on the interaction between skeletal muscle cells stimulated with palmitic acid (PA) and fibroblasts. We found that PA‐treated skeletal muscle cells actively secreted interleukin‐1β (IL‐1β) and augmented the migration, proliferation and expression of fibronectin in L929 fibroblasts. Furthermore, T‐CM inhibited the skeletal muscle cell‐derived pro‐fibrogenic effect *via* the production of the interleukin‐1 receptor antagonist (IL‐1Ra), which is an inhibitor of IL‐1 signalling. Taken together, our data provide novel insights into the therapeutic potential of T‐MSC‐mediated therapy for the treatment of pathophysiological processes that occur in skeletal muscle tissues under chronic inflammatory conditions.

## Introduction

Obesity has been largely associated with chronic low‐grade inflammation that is accompanied by excessively high plasma levels of free fatty acids (FFAs). Although the association of FFAs with insulin resistance in skeletal muscle is well known 1, 2, the inflammatory outcome, including pathophysiological fibrosis in muscle tissue in response to FFAs, have not yet been elucidated. In general, efficient muscle regeneration following injury requires the migration and proliferation of fibroblasts to promote the production of new temporary extracellular matrix (ECM) components, including collagen and fibronectin, which serve to stabilize the tissue and act as a scaffold for new fibres 3. However, disturbance of the fibrogenic process results in excessive deposition of connective tissue that progressively remodels, destroys and replaces the normal tissue architecture. Pathophysiological fibrosis is the result of a cascade of events that ensue as a result of chronic inflammation following tissue injury. Furthermore, fibrosis interferes with muscle regeneration, causes a loss of muscle function and alters the tissue environment, causing increased susceptibility to reinjury 4, 5. In cases of obesity, the most abundant FFA in the blood is palmitic acid (PA) 6. PA is a natural dietary ligand for toll‐like receptor 2 (TLR2) and TLR4 signalling, which ultimately leads to nuclear factor kappaB (NF‐κB) activation that promotes the production of pro‐inflammatory cytokines 7, 8. Thus, we assumed PA might function as a chronic inflammatory mediator that stimulates skeletal muscle tissue and causes an imbalanced interplay with fibroblasts. In fact, the muscle wasting and dysfunction that occurs in obesity 9, 10 indicates a possible link between inflammation and tissue deconstruction in skeletal muscle. Therefore, balancing the interplay of muscle‐connective tissue could be an efficient therapeutic option for treating diseases dominated by chronic inflammatory conditions, including obesity.

Mesenchymal stem cells (MSCs) are multipotent adult stem cells that have been shown to possess immunomodulatory and tissue regeneration properties which, in conjunction with their low immunogenic potential, make them a promising new treatment for numerous diseases 11. We previously reported that human palatine tonsil‐derived mesenchymal stem cells (T‐MSCs) were an attractive cellular source for immune modulation and tissue regeneration. To this end, exogenously administered T‐MSCs migrate to damaged tissue sites and participate in tissue repair by immune regulatory functions in a mouse model of liver injury 12, 13.

In the present study, we investigated whether PA‐treated muscle cells activated fibroblasts, and assessed the therapeutic effects of conditioned medium from T‐MSCs (T‐CM) on the interaction of skeletal muscle cells and fibroblasts. Finally, to obtain a better understanding of the immunomodulatory mechanisms of T‐CM, we attempted to identify crucial mediators by which T‐CM modulated muscular fibrosis.

## Materials and Methods

### Cell culture

C2C12 mouse myoblasts were kindly provided by Dr. Song (Dongguk University, Gyeongju, Korea) and cultured in growth medium consisting of Dulbecco's Modified Eagle Medium (DMEM; Welgene, Daegu, Korea) supplemented with 20% foetal bovine serum (FBS, Welgene), 100 U/ml penicillin and 100 μg/ml streptomycin at 37°C in a humidified atmosphere of 5% CO_2_. When the cells were confluent, the myoblasts were induced to differentiate into myotubes by changing the medium to DMEM containing 2% horse serum (HS, Sigma‐Aldrich, St. Louis, MO, USA), 100 U/ml penicillin and 100 μg/ml streptomycin. The differentiation medium was changed every 24 hrs and the differentiated cells (on days 5–7) were used for subsequent experiments.

The L929 mouse fibroblast cell line was purchased from the Korean cell line bank (No. NCTC clone 929, Seoul, Korea) and cultured in Roswell Park Memorial Institute (RPMI; Welgene, Daegu, Korea) medium supplemented with 10% FBS, 100 U/ml penicillin and 100 μg/ml streptomycin at 37°C in a humidified atmosphere of 5% CO_2_.

### Haematoxylin and eosin stain of C2C12 myotubes

C2C12 cells were seeded in the 2 well chamber slide (Nunc, NY, USA) at a density of 8 × 10^4^ cells per well. On the next day, the medium were changed with DMEM containing 2% HS, 100 U/ml penicillin and 100 μg/ml streptomycin for differentiation into myotubes. After 7 days, cells were fixed with 100% methanol and stained with haematoxylin and eosin (H&E) to observe the morphological changes upon differentiation.

### Reverse transcription polymerase chain reaction (RT‐PCR)

For the analysis of TLR expression in C2C12 myoblasts and differentiated myotubes, the cells were harvested and homogenized in TRIzol^®^ (Invitrogen, MA, USA). Mouse brain and spleen tissues were collected from 8 week old female C57BL/6 mouse and conducted as negative control and positive control to compare the expression of TLRs, respectively. Moreover, the expression of TLRs on myotubes supplemented with conditioned medium from T‐MSC for 24 hrs on day 6 were investigated to compare with those of myotubes non‐treated.

Complementary DNA was synthesized using the First‐Strand cDNA Synthesis Kit (Toyobo, Osaka, Japan) according to the manufacturer's instructions. The primers used in the reaction were obtained in public resource for PCR primers, PrimerBank (https://pga.mgh.harvard.edu/primerbank/) and In‐Silico PCR was confirmed in UCSC genome browser (https://genome.ucsc.edu/). The reaction was amplified using the following primers; 5′‐TGTCCCAACTACAGTTCCTGG‐3′ (forward) and 5′‐TGCTAACGTGCCGAAGAGATT‐3′ (reverse) for Tlr1 (153 bp); 5′‐CTCTTCAGCAAACGCTGTTCT‐3′ (forward) and 5′‐GGCGTCTCCCTCTATTGTATTG‐3′ (reverse) for Tlr2 (237 bp); 5′‐TGCCAAATACTCCCTTTGTTGAA‐3′ (forward) and 5′‐CCCGTTCCCAACTTTGTAGATG‐3′ for Tlr3 (218 bp); 5′‐ATGGCATGGCTTACACCACC‐3′ (forward) and 5′‐GAGGCCAATTTTGTCTCCACA‐3′ (reverse) for Tlr4 (129 bp); 5′‐TGGGGACCCAGTATGCTAACT‐3′ (forward) and 5′‐CCACAGGAAAACAGCCGAAGT‐3′ (reverse) for Tlr5 (160 bp); 5′‐TGAGCCAAGACAGAAAACCCA‐3′ (forward) and 5′‐GGGACATGAGTAAGGTTCCTGTT‐3′ (reverse) for Tlr6 (139 bp); 5′‐ATGTGGACACGGAAGAGACAA‐3′ (forward) and 5′‐GGTAAGGGTAAGATTGGTGGTG‐3′ (reverse) for Tlr7 (207 bp); 5′‐GCCAAACAACAGCACCCAAAT‐3′ (forward) and 5′‐AGGCAACCCAGCAGGTATAGT‐3′ (reverse) for Tlr8 (132 bp); and 5′‐ATGGTTCTCCGTCGAAGGACT‐3′ (forward) and 5′‐ CAGGTGGTGGATACGGTTGG‐3′ (reverse) for Tlr9 (234 bp). Glyceraldehyde 3‐phosphate dehydrogenase (*Gapdh*) was used as the internal control gene and was amplified using the primers 5′‐GGTAAAGTGGATATTGTTGCCATCAATG‐3′ (forward) and 5′‐GGAGGGATCTCGCTCCTGGAAGATGGTG‐3′ (reverse) (173 bp). The band pixel densities of genes were divided by the pixel densities of the corresponding Gapdh bands for quantitation using UN‐SCAN‐IT‐gel 6.1 software (Silk Scientific, Inc., Orem, UT, USA).

### Quantitative reverse transcription PCR (qRT‐PCR)

For the analysis of fibronectin expression in L929 cells, complementary DNA was synthesized using the First‐Strand cDNA synthesis kit (Toyobo) according to the manufacturer's instructions. RT‐PCR analysis was performed on a StepOnePlus^™^ instrument (Applied Biosystems, Carlsbad, CA, USA) using SYBR^®^ green (Toyobo). Fibronectin (124 bp) was amplified using the primers, 5′‐ATGTGGACCCCTCCTGATAGT‐3′ (forward) and 5′‐GCCCAGTGATTTCAGCAAAGG‐3′ (reverse). The expression of fibronectin was normalized to the expression of mouse Gapdh and Relative fold expression and changes were calculated using the 2^−ΔΔCt^ method**.**

### Preparation of conditioned medium

To generate MSC‐conditioned medium (MSC‐CM), BM‐derived MSCs (BM‐MSCs), adipose tissue‐derived MSCs (AT‐MSCs) and T‐MSCs (at passages 7–8) were grown to 80–90% confluence in 100‐mm tissue culture plates. The T‐MSCs were obtained and maintained as previously reported 14. The AT‐MSCs were generously provided by RNL Bio (Seoul, Korea), and the BM‐MSCs were purchased from the Severance Hospital Cell Therapy Center (Seoul, Korea). At 80–90% confluence, the cells were washed twice with phosphate‐buffered saline, and the medium was replaced with serum‐free DMEM to generate the CM. The medium was collected after 48 hrs of culture, centrifuged at 1300 rpm for 5 min. and passed through a 0.2‐μm filter. The CM was concentrated 20‐fold by centrifugal filtration (3K cut‐off; Amicon Ultra‐15, Millipore, MA, USA). The concentrated CM was frozen and stored at −80°C for future use. Serum‐free culture medium was processed in the same manner to serve as a negative control.

### Palmitate treatment

Palmitic acid (Sigma‐Aldrich) was conjugated with fatty acid‐free bovine serum albumin (BSA, Bovogen Biologicals, East Keilor, VIC, Australia). Briefly, FFAs were dissolved in ethanol and added to serum‐free DMEM containing 2% BSA at a concentration of 7.5 mM. The medium was then incubated at 37°C for 2 hrs on a stirrer, filtered and used to treat the C2C12 myotubes. The C2C12 myotubes were treated with PA at a final concentration of 750 μM in the presence or absence of T‐CM. The T‐CM was supplemented with an equal ratio of T‐MSCs and myoblasts based on confluent cell numbers. For example, when 600 μl of T‐CM was derived from 2.4 × 10^6^ T‐MSCs (the number when it reached confluence in 100‐mm culture plates), 150 μl of T‐CM from the total 600 μl was used to treat 6 × 10^5^ C2C12 myotubes.

### Transfection

To reduce endogenous interleukin‐1 receptor antagonist (IL‐1Ra) expression, the T‐MSCs were transfected with IL‐1Ra‐specific siRNA oligonucleotides (Santa Cruz Biotechnology, Santa Cruz, CA, USA) using Lipofectamine^®^ 2000 reagent (Thermo Fisher Scientific, Waltham, MA, USA) in accordance with the manufacturer's instructions. Non‐targeted siRNA oligonucleotides (Santa Cruz Biotechnology) were used as negative controls. At 48 hrs post‐transfection, the cells were harvested for protein extraction and 10 μg of protein was used to confirm IL‐1Ra knockdown by western blotting.

### Western blotting

Cell culture supernatant from the control C2C12 myotubes, T‐CM‐treated C2C12 myotubes, PA (750 μM)‐treated C2C12 myotubes and T‐CM plus PA‐treated C2C12 myotubes was collected to detect interleukin‐1 beta (IL‐1β) secretion into the culture supernatant. An equal amount of supernatant was loaded in each lane, and after blotting, the membranes were incubated overnight with a primary antibody against IL‐1β (Santa Cruz Biotechnology, H‐153). After intensive washing, the membranes were incubated with the corresponding secondary antibody (antimouse IgG, Sigma‐Aldrich) and detected using an enhanced chemiluminescence reagent (Thermo Fisher Scientific). To normalize the secreted IL‐1 β, TGF‐β in the same cell supernatant was detected by using antibody against TGF‐β (Santa Cruz Biotechnology, H‐112). The band pixel densities of IL‐1 β were divided by the pixel densities of the corresponding TGF‐β bands for quantitation using UN‐SCAN‐IT‐gel 6.1 software (Silk Scientific, Inc.).

On the other hand, equal amounts (20 μl per lane) of CM from the different types of MSCs (BM‐MSCs, AT‐MSCs, and T‐MSCs) were subjected to western blotting to compare the extent of IL‐1Ra released into the cell culture supernatant as described above. The expression of IL‐1Ra was detected by using antibody against IL‐1Ra (Santa Cruz Biotechnology, H‐110). The extent of other releasing protein, PD‐L2, in CM derived from the different types of MSCs was also determined by western blot using antibody against PD‐L2 (Santa Cruz Biotechnology, H‐67). The band pixel densities of IL‐1Ra were divided by the pixel densities of the corresponding PD‐L2 bands for quantitation using UN‐SCAN‐IT‐gel 6.1 software (Silk Scientific, Inc.). In addition, 800 ng of recombinant IL‐1Ra (rIL‐1Ra, Biolegend) was loaded separately to quantitate concentration of secreted IL‐1Ra in T‐CM. 20 μl of T‐CM contains approximately 100 ng of IL‐1Ra when quantitate the band pixel densities using UN‐SCAN‐IT‐gel 6.1 software (Silk Scientific, Inc.).

The endogenous levels of IL‐1Ra in T‐MSCs were also investigated, and a primary antibody against β‐actin (Sigma‐Aldrich) was used for the normalization of IL‐1 Ra expression as described above.

For the analysis of fibronectin expression in L929 cells, the cellular levels of fibronectin were detected using antibodies against fibronectin (Santa Cruz Biotechnology, EP5). The band pixel densities of fibronectin were divided by the pixel densities of the corresponding β‐actin bands for normalizing using UN‐SCAN‐IT‐gel 6.1 software (Silk Scientific, Inc.).

### Transwell migration assay

Transwell cell migration assays were performed with Transwell^®^‐24 well permeable support plates with an 8‐μm pore size polycarbonate membrane (Corning, NY, USA). L929 cells (10^5^) were placed on the upper layer of the cell‐permeable membrane in serum‐free DMEM. As a negative control, serum‐free DMEM without C2C12 myotubes was added to the bottom of the support plate. DMEM containing 2% HS without the C2C12 myotubes was likewise tested. DMEM supplemented with rIL‐1β (100 ng/ml) and 2% HS was added to the bottom of the support plate as a positive control for the migration of L929 cells. T‐CM or C2C12 myotubes were added to the bottom of the support plate to serve as a comparison of the basal effects on L929 migration. The treated volume of T‐CM is 20 μl and this approximately works at a final concentration of 100‐140 ng/ml for IL‐1Ra activity according to our quantitation of IL‐1Ra in T‐CM by performing western blot.

To observe the chemotactic activity of PA pre‐treated C2C12 myotubes, the myotubes were seeded in the lower chamber. T‐CM from IL‐1Ra knockdown T‐MSCs or normal control T‐MSCs, as well as rIL‐1Ra, were added to separate chambers containing C2C12 myotubes pre‐treated with PA. Following a 10 hrs incubation, the cells that migrated through the pores onto the lower side of the membrane were fixed in 70% ethanol for 10 min., air‐dried for 10 min. and stained with 0.2% crystal violet for 10 min. The insert was then carefully washed three times with distilled water, air‐dried, and the cells were counted using a phase‐contrast microscope.

### Cell proliferation assay

Before seeding for co‐culture with C2C12 myotubes, L929 cells were labelled with carboxyfluorescein diacetate succinimidyl ester (CFSE, CellTrace^™^ CFSE Cell Proliferation kit, Invitrogen, Paisley, United Kingdom) in accordance with the manufacturer's instructions.

After CFSE labelling, 10^5^ cells were placed in the bottom of a Transwell^®^‐24 well permeable support plate (Corning) with a 3 μM pore insert (Corning) that contained either C2C12 myotubes pre‐treated overnight with PA or untreated C2C12 myotubes in the upper part. As a positive control, recombinant IL‐1β (rIL‐1β, 100 ng/ml, PeproTech, Rocky Hill, NJ, USA) was added to the bottom of the permeable support plate in the absence of myotubes. To confirm the effect of T‐CM on the induction of fibroblast proliferation by the myotubes, T‐CM (20 μl) was added to the bottom of the co‐culture system. Recombinant IL‐1Ra (rIL‐1Ra, 100 ng/ml, PeproTech) was used as a comparison to the effects of T‐CM. After 3 days, the degree of proliferation of the CFSE positive cells was measured by flow cytometry and analysed using the ModFit LT^™^ software (Verity Software House, Topsham, ME, USA), based on the reduction in CFSE positive cells.

### Statistical analysis

Data are presented as means ± standard error of the mean (SEM). Statistical significance was determined by One way anova or Two way anova using the GraphPad Prism 6 software (GraphPad Software Inc., San Diego, CA, USA). For all analyses, *P* < 0.05 was considered statistically significant.

## Results

### TLR2, TLR3 and TLR4 are highly expressed in C2C12 myoblasts and C2C12 myotubes

**Figure 1:**
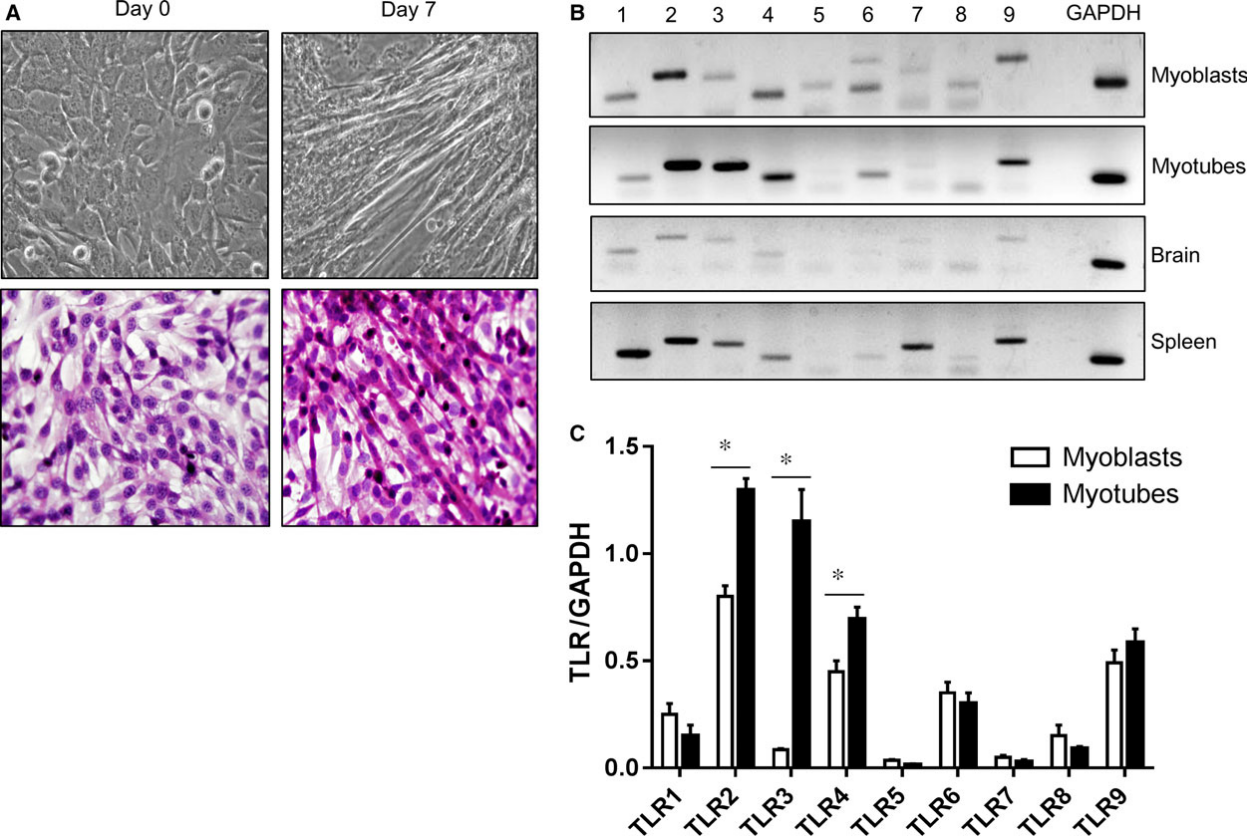
C2C12 myoblasts and myotubes constitutively express TLRs. (**A**) C2C12 myoblasts were fully differentiated by day 7 and morphological changes were observed by phase contrast microscope (upper panel, Original magnification, 400×). Cells were fused to form multinucleated myotubes as shown in haematoxylin and eosin stain (lower panel, Original magnification, 400×). (**B**) C2C12 myoblasts and myotubes were harvested and the expression of TLR1–TLR9 was detected by RT‐PCR. TLR2, TLR3 and TLR4 were highly induced upon differentiation. The expression of TLRs on mouse brain and spleen tissues were compared as negative control and positive control, respectively. (**C**) The pixel densities of the TLR amplicon bands were divided by the pixel densities of the corresponding *Gapdh* bands. The data are presented as the mean ± SEM (*P < 0.05). TLR, toll‐like receptor.

PA, a saturated FFA, acts as a ligand for TLR2 and TLR4, and induces a series of inflammatory cascade‐related responses 7, 8. Thus, we tested whether C2C12 myoblasts and myotubes expressed TLR2 and TLR4 at the steady‐state. C2C12 myoblasts started to form myotubes on day 2–3 under differentiation conditions and were fully differentiated by day 7 (Fig. 1A). Both myoblasts and myotubes constitutively expressed TLR 1 to TLR 9; however, TLR2, TLR3, TLR 4, in particular, were significantly increased upon differentiation (Fig. 1B–C), which implies that skeletal muscle cells effectively sense PA.

### C2C12 myotubes secrete the pro‐inflammatory cytokine IL‐1β, and T‐MSCs produce IL‐1Ra

**Figure 2:**
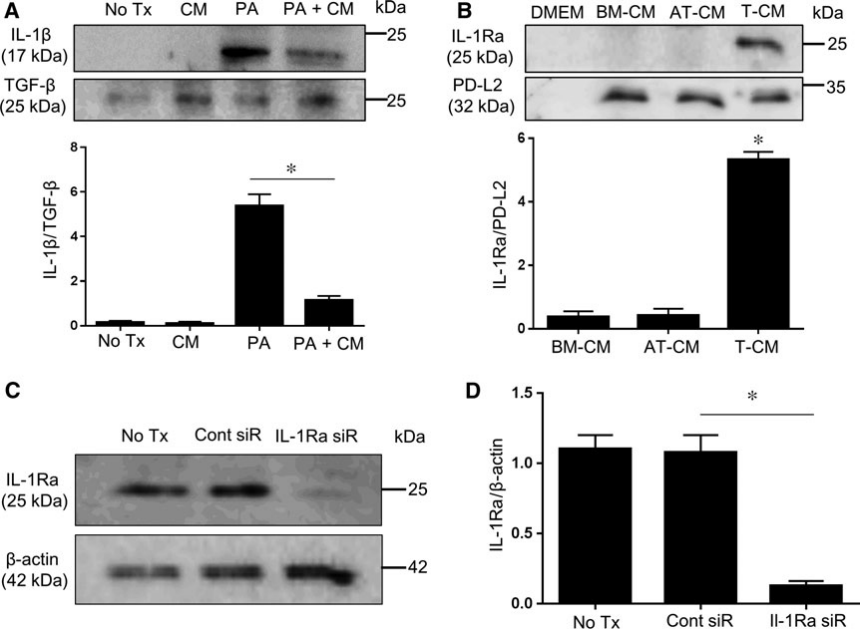
PA‐treated C2C12 myotubes release IL‐1β, and T‐MSCs constitutively produce IL‐1Ra. (**A**) C2C12 myotubes were treated with 750 μM of PA in the absence or presence of T‐CM for 24 hrs and the cell culture supernatants were collected. The expression of IL‐1β was observed by western blotting using the cell culture supernatant. Supernatant from untreated cells and cells treated with T‐CM only were likewise compared. The other releasing factor, TGF‐β, in cell culture supernatant from each experimental group was detected and compared for normalization of secreted IL‐1β. The pixel densities of the IL‐1β bands were divided by those of TGF‐β bands for normalization. The data are presented as the mean ± SEM (*P < 0.05). (**B**) Cell culture supernatants were collected and subjected to western blotting to detect secreted IL‐1Ra in BM‐MSCs, AT‐MSCs, and T‐MSCs. The extent of secreted PD‐L2 in supernatant from each MSCs was determined and used as equivalent releasing factors for normalizing IL‐1Ra. The pixel densities of the IL‐1Ra bands were divided by those of corresponding PD‐L2 bands for normalization. The data are presented as the mean ± SEM (*P < 0.05). (**C**) *Il‐1ra*‐specific siRNA effectively knocked down endogenous IL‐1Ra expression in T‐MSCs. (**D**) The pixel densities of the IL‐1Ra bands were divided by those of the corresponding β‐actin bands for normalization. The data are presented as the mean ± SEM (*P < 0.05).

We previously reported that excessive lipid accumulation within skeletal muscle cells as a result of overexpressing lipid droplets induces IL‐1β production *via* activation of the NLRP3 inflammasome pathway 15. Thus, we speculated that exogenous treatment with PA might also stimulate IL‐1β secretion by C2C12 myotubes. As shown in Figure 2A, PA‐treated myotubes released marked levels of IL‐1β into the cell culture supernatant. Treatment with T‐CM alone did not affect the production of IL‐1β by myotubes. However, PA‐treated myotubes secreted reduced levels of IL‐1β in the presence of T‐CM, most likely because of a downregulating effect of T‐CM on TLR expression in the myotubes (Fig. S1).

As we showed IL‐1β was highly produced in myotubes only when exposed to PA, we tested whether other releasing factors are influenced by PA as well. TGF‐β is one of the secretory proteins from myotubes 16, we compared the extent of secreted TGF‐β in the same cell supernatant. In contrast with IL‐1β, we confirmed TGF‐β was released from all experimental cell supernatant with tendency of slight increase in the presence of T‐CM. Thus, IL‐1β is critical production of PA exposed myotuebs.

On the basis of this finding, we hypothesized that skeletal muscle cells may activate fibroblasts by IL‐1β stimulation. Therefore, we tried to find the modulatory mediator in T‐MSCs that effectively blocked the activity of IL‐1β derived from C2C12 myotubes. The protein of interest was IL‐1Ra, which is a natural inhibitor of the pro‐inflammatory effects of IL‐1β.

Because IL‐1R is the same receptor that binds IL‐1 (IL‐1α and IL‐1β), the binding of IL‐1Ra prevents IL‐1 from binding and sending a signal to the cell 17. We found that T‐MSCs constitutively produce IL‐1Ra because the T‐MSC cell culture supernatant contained IL‐1Ra, whereas the culture supernatant from the other types of MSCs (BM‐MSCs and AT‐MSCs) did not (Fig. 2B). To normalize secreted IL‐1Ra, we investigated other releasing factors from each conditioned medium. PD‐L2, is a member B7 family members and is ligand for PD‐1. By ligation with PD‐1 on immune cells, in particular T cells, PD‐L2 initiate inhibitory signals to regulate T cell differentiation or T cell mediated immune response 18. As shown in Figure 2B, PD‐L2 was uniformly secreted from BM‐MSCs, AT‐MSCs, and T‐MSCs. This indicate IL‐1Ra is specific mediator derived from T‐MSCs. T‐CM approximately contain 100 ng of IL‐1Ra within 20 μl volume as revealed in quantitation by normalizing with band pixel densities of 800 ng loaded rIL‐1Ra (Fig. S2).

Prior to investigating the effects of T‐MSC‐derived IL‐1Ra, we knocked down the endogenous expression of IL‐1Ra in T‐MSCs by transfecting the cells with IL‐1Ra‐specific siRNA (Fig. 2C–D). The expression of IL‐1Ra was reduced up to 90% by siRNA transfection.

### T‐CM suppresses the migration of fibroblasts induced by PA‐stimulated C2C12 myotubes *via* IL‐1Ra activity

**Figure 3:**
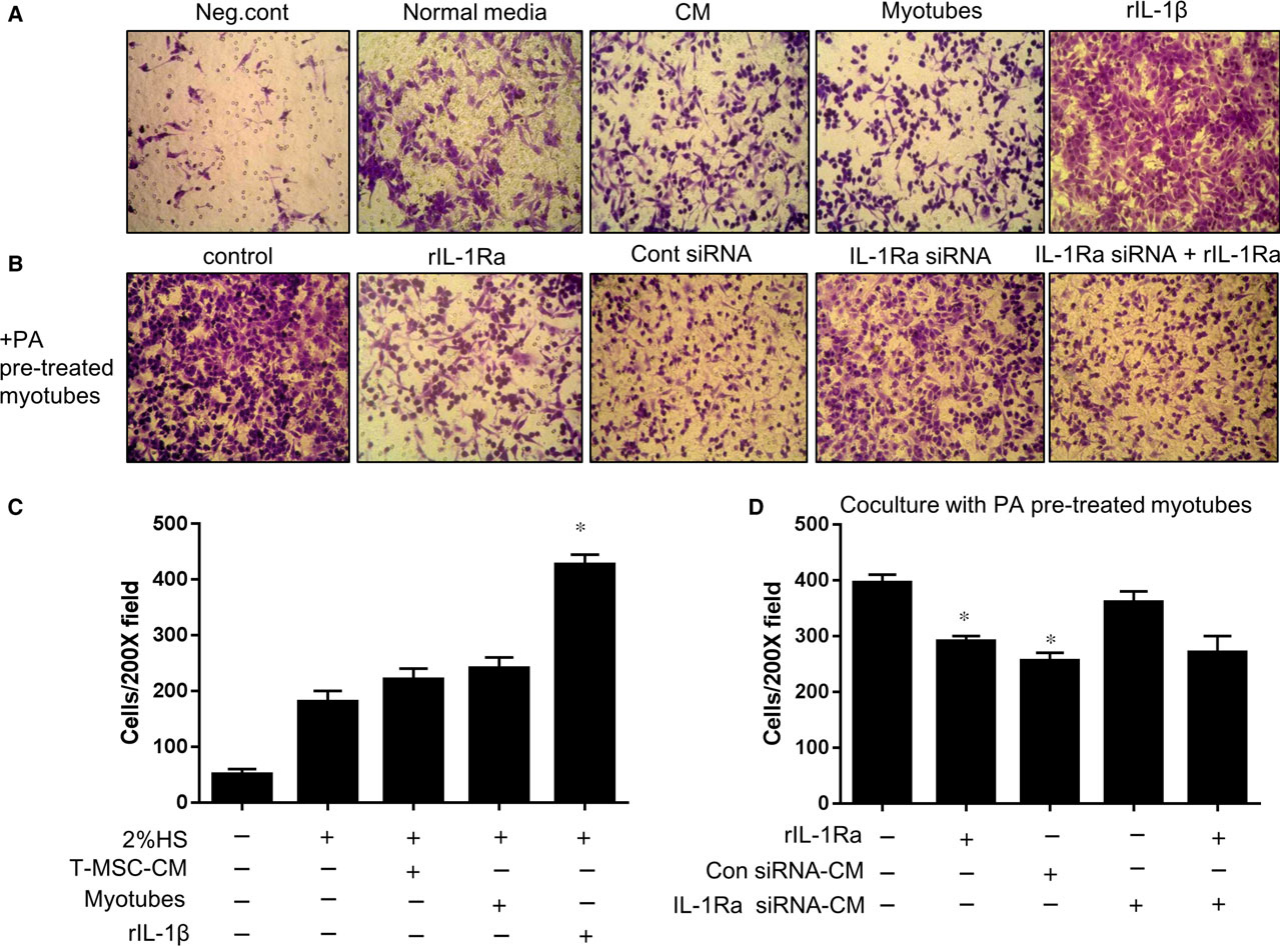
The migration of L929 fibroblasts following exposure to PA‐stimulated myotubes was abrogated by T‐CM in an IL‐1Ra‐dependent manner. (**A**) L929 cells were placed in the upper chamber of a transwell plate to test the ability to migrate through the membrane. In the bottom chamber, DMEM containing 2% HS was added as a normal condition. T‐CM or myotubes were placed in the bottom chamber to confirm their basal ability to recruit L929 cells. The placement of DMEM without serum or DMEM with 2% HS and 100 ng/ml of rIL‐1β in the bottom chamber were tested as the negative and positive controls, respectively. After 10 hrs, L929 cells that passed through the insert membrane and adhered to the opposite side were stained with crystal violet solution. Original magnification, 200×. (**B**) To confirm the effect of PA‐treated myotubes on the migration of L929 cells, myotubes were pre‐treated with 750 μM PA overnight in the bottom chamber and the media was changed after the addition of L929 cells in the upper chamber. rIL‐1Ra or T‐CM from T‐MSCs transfected with either *Il‐1ra‐*specific siRNA or control siRNA were added to the myotubes to test the correlation of the IL‐1β derived from myotubes and the migration of L929 cells. Exogenous rIL‐1Ra treatment (100 ng/ml) in the lower chamber containing T‐CM derived from IL‐1Ra knockdown T‐MSCs was tested as well. Original magnification, 200×. (**C**) L929 cells that migrated through the pores onto the lower side of the membrane were fixed, stained and counted using a phase‐contrast microscope. The data are presented as the mean ± SEM (*P < 0.05). (**D**) L929 cells migrated toward PA pre‐treated myotubes on the bottom in the plate were fixed, stained and counted using a phase contrast microscope. The data are presented as mean ± SEM (*P < 0.05)

Fibroblasts are capable of directed migration in response to pro‐inflammatory cytokines released at sites of injury, which is a key step in wound healing 19. IL‐1β has been recognized as a potent pro‐inflammatory cytokine that enhances cell migration under inflammatory conditions 20, 21. Thus, we tested the effect of PA pre‐treated myotubes on the migration of L929 cells *via* a transwell migration assay. The L929 cells in the upper insert did not migrate in response to T‐CM or myotubes, which were placed in the bottom of the plate; however, rIL‐1β strongly induced the migration of the cells through the membrane pores (Fig. 3A). Because we observed that myotubes secreted IL‐1β when exposed to PA, myotubes pre‐treated with PA were tested to determine whether PA induced the migration of L929 cells. To control for the direct effects of PA on L929 cells, the myotubes were stimulated with PA overnight and the media was changed before the addition of the insert containing the L929 cells. Figure 3B shows that PA‐stimulated myotubes enhanced the migration of L929 cells, and that the addition of the IL‐1Ra inhibitor reversed the phenomenon. We previously observed that rIL‐1Ra effectively inhibit migration of L929 cells towards PA‐stimulated myotubes in a dose dependent manner (Fig. S3). Of note, T‐CM effectively reduced the migratory ability of L929 cells, possibly *via* the production of IL‐1Ra, as shown by the reduced effects of the T‐CM derived from IL‐1Ra knockdown T‐MSCs. Furthermore, the decreased effects of the T‐CM were offset by the addition of exogenous rIL‐1Ra.

### T‐CM inhibits fibroblast proliferation induced by PA‐stimulated C2C12 myotubes *via* IL‐1Ra activity

**Figure 4:**
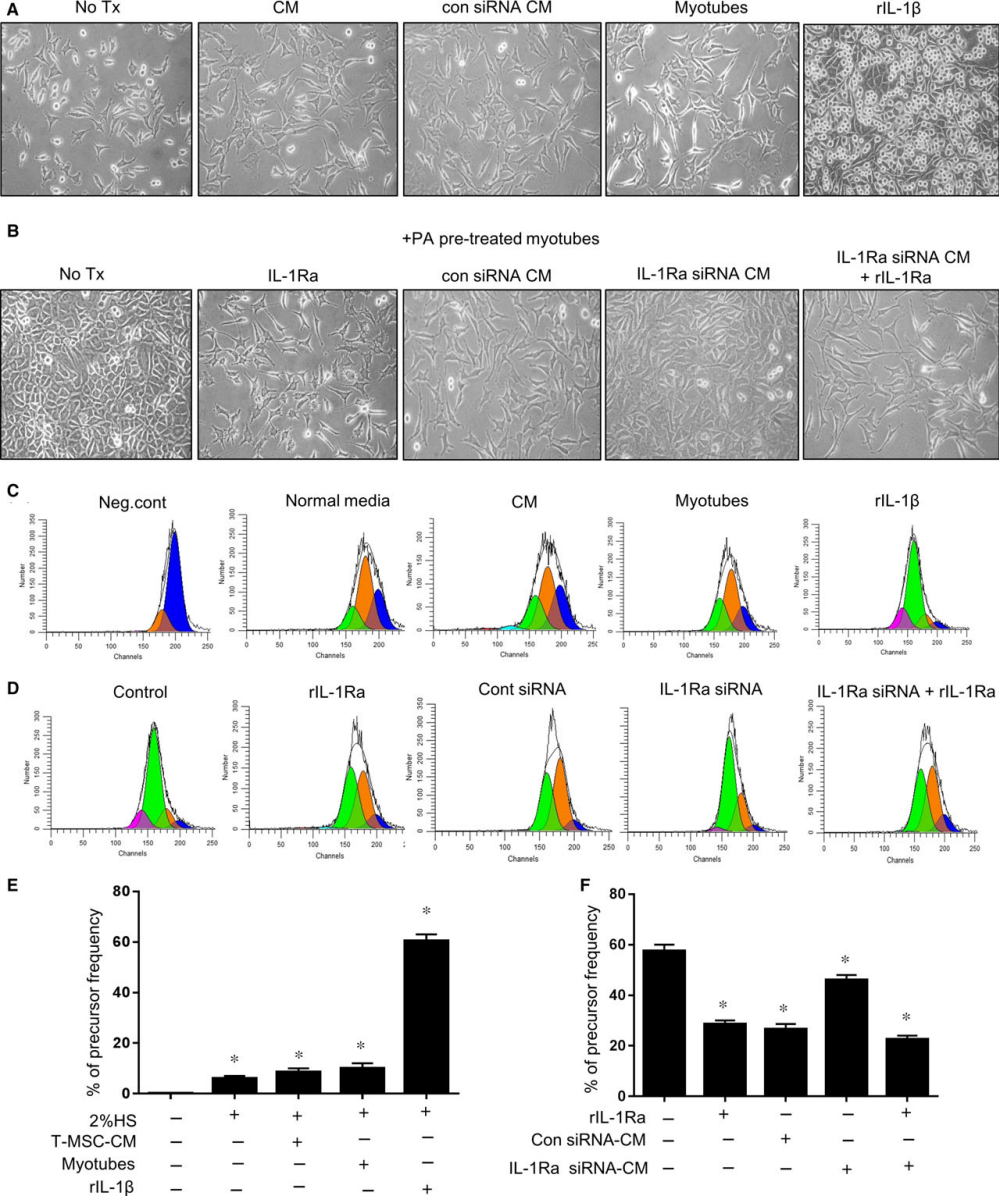
Proliferation of L929 fibroblasts in the exposure to PA‐stimulated myotubes was attenuated by T‐CM containing IL‐1Ra. (**A**) L929 cells labelled with CFSE were seeded in the bottom of a 24‐well transwell plate. T‐CM or T‐CM from control siRNA‐transfected T‐MSCs was added to the media to test their basal activity on the proliferation of L929 cells. In the upper insert, myotubes or rIL‐1β (100 ng/ml) were added, respectively. The proliferated L929 cells were monitored using a phase‐contrast microscope after culturing for 3 days. Original magnification, 200×. (**B**) To confirm the effect of PA‐treated myotubes on the proliferation of L929 cells, myotubes pre‐treated with PA were placed in the upper chamber in new media. rIL‐1Ra (100 ng/ml) or T‐CM from T‐MSC transfected with either IL‐1Ra‐specific siRNA or control siRNA was supplemented to the lower chamber containing L929 cells. In addition, L929 cells in the bottom chamber were treated with a combination of rIL‐1RA (100 ng/ml) and T‐CM from T‐MSCs transfected with *Il‐1ra*‐specific siRNA. Microscopic observations are presented. Original magnification, 200×. (**C–F**) After culturing for 3 days, L929 cells in the lower chamber were collected. CFSE+ cells were measured for the degree of proliferation *via* flow cytometry and were analysed using the ModFit LT ^™^ software based on the reduction in CFSE positive cells. Result presented in (**D**) and (**F**) is from cells exposed to PA pre‐treated myotubes that located in the upper chamber. The data are presented as the mean ± SEM (*P < 0.05).

Once fibroblasts are recruited to the skeletal muscle tissue, the local proliferation of fibroblasts ensues during the process of fibrosis. In addition, the persistent secretion and subsequent deposition of excessive ECM is a critical feature of fibrosis 22. Nonetheless, the direct effects of the release of IL‐1β by muscle cells on fibroblast activation, including proliferation and ECM expression, have not yet been elucidated. We compared the extent of L929 cell proliferation in a transwell co‐culture system. As shown in Figure 4, L929 cells placed in the lower chamber of the co‐culture system exhibited differential proliferation in accordance with various experimental conditions. Supplementation with T‐CM or T‐CM from control, non‐specific siRNA‐transfected T‐MSCs did not affect the proliferation of L929 cells. Furthermore, L929 cells were maintained under normal conditions during exposure to non‐treated myotubes, which were placed in the upper chamber of the co‐culture system. However, rIL‐1β and myotubes pre‐treated with PA augmented the proliferation of L929 cells, most likely as a result of the direct activity of IL‐1β, which was supported by the normalized proliferation apparent in the rIL‐Ra treatment assay. Hence, the T‐CM from the IL‐1Ra knockdown T‐MSCs showed weaker effects on the regulation of L929 cell proliferation, but supplementation with rIL‐1Ra rescued the activity.

### T‐CM‐derived IL‐1Ra decreases fibronectin expression by L929 cells under PA‐stimulated C2C12 myotubes

**Figure 5:**
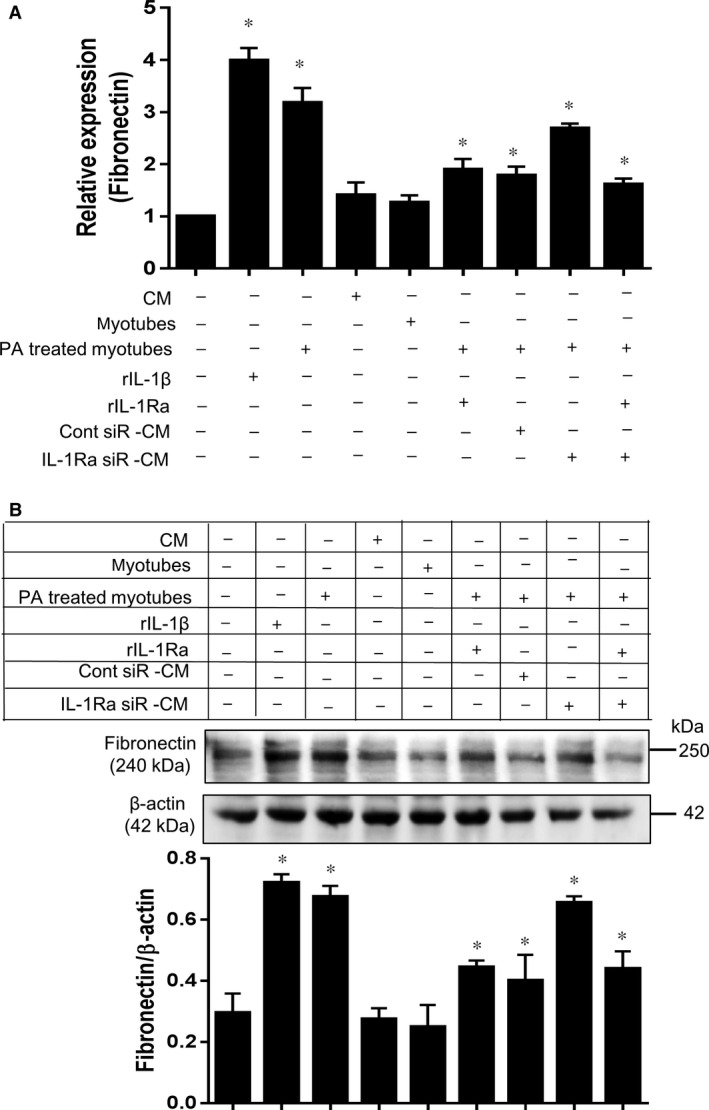
T‐CM inhibits fibronectin expression in L929 cells induced by PA‐stimulated myotubes in an IL‐1Ra‐dependent manner. (**A**) L929 cells, cultured under various conditions as indicated, were harvested and the expression of fibronectin was quantified by RT‐PCR. The co‐culturing of L929 cells with myotubes was performed in a 24‐well transwell plate. The L929 cells were placed in the lower chamber and the myotubes were seeded in the upper chamber. rIL‐1β (100 ng/ml), experimental T‐CM, and rIL‐1Ra (100 ng/ml) were added to the bottom chamber, which contained the L929 cells. The data are presented as the mean ± SEM (*P < 0.05). (**B**) The protein level of fibronectin was determined by western blot in the same experimental group. The pixel densities of the fibronectin bands were divided by those of the corresponding β‐actin bands for normalization. The data are presented as the mean ± SEM (*P < 0.05).

Fibronectin is a ubiquitous ECM glycoprotein that plays vital roles in tissue repair 23. Furthermore, excessive deposition of ECM, of which fibronectin is one of the major components, occurs during fibrosis. Aberrant fibronectin matrix assembly is a major contributing factor in the switch from normal tissue repair to misregulated fibrosis. A recent study demonstrated that blocking fibronectin deposition results in decreased collagen accumulation and improved liver function during liver fibrogenesis 24. Thus, we speculated that the mediation of the fibrotic process by skeletal muscle might involve the induction of fibronectin expression in fibroblasts. IL‐1β or PA pre‐treated myotubes significantly induced the expression of fibronectin either RNA and protein level in L929 fibroblasts in a transwell co‐culture system, whereas the expression pattern following exposure to normal control T‐CM or myotubes was intact (Fig. 5A–B). Notably, T‐CM significantly downregulated the expression of fibronectin in L929 cells even in the presence of PA pre‐treated myotubes and was as effective as rIL‐1Ra treatment. Finally, rIL‐1Ra supplementation rescued the altered activity of T‐CM in IL‐1Ra knockdown T‐MSCs and regulated fibronectin expression in L929 cells.

## Discussion

In this study, we demonstrated that T‐CM regulated the interaction between PA‐treated C2C12 myotubes and the L929 fibroblast cell line. C2C12 myotubes stimulated with PA secreted excessive levels of the IL‐1β pro‐inflammatory cytokine and subsequently recruited, promoted hyperproliferation and induced fibronectin expression in L929 cells. Importantly, we observed that T‐MSCs constitutively expressed and secreted IL‐1Ra, which is a receptor antagonist for the IL‐1 receptor. Thus, T‐CM efficiently regulated the mediation of the myotubes' pro‐fibrogenic process by altering the IL‐1β activity of myotubes.

MSCs are multipotent adult stem cells that can differentiate into a variety of cell types. The therapeutic effects of MSCs are believed to occur not only by direct differentiation in injured tissue, but also by the production of paracrine factors that inhibit apoptosis, stimulate endogenous cell proliferation, and/or activate resident stem cells at the site of injury. Moreover, the discovery that MSCs contribute to tissue regeneration by modulating inflammation has revolutionized stem cell therapy for the treatment of inflammatory diseases 25. A chronic inflammatory status eventually causes the destruction of target organ tissue in accordance with disease 26. Therefore, cell therapy using MSCs or its conditioned medium could be an effective therapeutic strategy for the treatment of many chronic inflammatory diseases that are accompanied by tissue injury. A mounting body of evidence has demonstrated a potential link between metabolic disorders (such as type 2 diabetes, obesity and atherosclerosis) and chronic low‐grade inflammation characterized by abnormal cytokine or lipid mediator production and the activation of a network of inflammatory signalling pathways in tissues targeted by insulin, including fat, liver and muscles 27. For example, excessively high plasma FFA levels are found in diabetic patients as well as in non‐diabetic subjects. Further, a high concentration of FFAs, particularly PA, which is the most abundant dietary saturated fatty acid, can directly impair insulin signalling and glucose uptake in skeletal muscle cells 28, 29. Thus far, studies of skeletal muscle have predominantly focused on defects in orchestrating insulin signals during obesity. Although it is recognized that inflammation is triggered by lipid‐laden adipose tissue under obese conditions, recent studies have demonstrated that skeletal muscle also secretes pro‐inflammatory cytokines, such as tumour necrosis factor alpha (TNF‐α), in response to elevated FFA levels 30. However, those findings were restricted to investigations of the mechanisms underlying insulin resistance. We noticed a potential link between inflammatory‐activated skeletal muscle tissue and neighbouring connective tissue containing fibroblasts. Pathophysiological fibrosis, which is essentially an excessive accumulation of ECM components, is the end result of a cascade of events that follow tissue injury 22. In general, a fibrotic reaction occurs following chronic tissue inflammation. Because fibrosis ultimately impairs tissue functions, regulating fibrogenic processes could be an important therapeutic option in diseases associated with chronic inflammation. In particular, skeletal muscle is the critical target organ that is continually responsive to circulating PA. Therefore, muscular fibrosis could potentially lead to muscle wasting and dysfunction. Herein, we aimed to first confirm whether skeletal muscle exposed to PA facilitated the pro‐fibrogenic process, and second, to determine the therapeutic effect of T‐CM and other relevant mediators of the observed effects.

PA is a natural dietary ligand for the activation of the TLR2 and TLR4 signalling pathways, which ultimately leads to NF‐κB activation in macrophages and the release of pro‐inflammatory cytokines 7, 8. Thus, we speculated that skeletal muscle cells could produce pro‐inflammatory mediators in response to PA. Previously, we found that C2C12 myoblasts produce IL‐1β following the accumulation of intracellular lipids by increasing lipid droplets within the cells 15. Therefore, we hypothesized that C2C12 myotubes were capable of producing IL‐1β following exposure to PA because FFAs cause intracellular lipid accumulation. C2C12 myoblasts expressing TLR2 and TLR4 were increased following differentiation and the cells exhibited increased IL‐β secretion in association with stimulation by PA. Of note, we confirmed that the effects of IL‐1β from myotubes were diminished when T‐CM was added in conjunction with the PA treatment, most likely because of the immunomodulatory mediators in T‐CM, such as TNF‐stimulated gene 6 protein (TSG‐6, data not shown), which is a known downregulator of TLR2/NF‐kB signalling 31. As expected, PA‐treated myotubes promoted the migration, proliferation, and expression of the ECM component fibronectin in L929 fibroblasts in a co‐culture system.

Although IL‐1β is closely associated with inflammation and subsequent tissue destruction in rheumatoid arthritis and periodontitis primarily by the acceleration of fibrosis 32, 33, the involvement of IL‐1β in pathologic fibrosis in muscle tissue has not yet been studied. Here, we first showed that skeletal muscle cells were active immune mediators that directly induced the pro‐fibrogenic process. Likewise, we demonstrated that T‐CM suppressed the pathophysiological interaction of myotubes and fibroblasts by IL‐1Ra activity. IL‐1Ra is a naturally occurring inhibitor of the IL‐1 receptor that competes with IL‐1α and IL‐1β for binding to IL‐1Ra and thus competitively inhibits IL‐1 activity 17. An IL‐1Ra deficiency in mice results in spontaneous and lethal arteritis, destructive arthritis and psoriatic‐like skin lesions, as well as increased susceptibility to carcinogenesis. Children born with a genetic IL‐1Ra deficiency or with functionally inactive IL‐1Ra suffer from severe systemic and local inflammation, including pustular skin eruptions, vasculitis, osteolytic lesions and sterile osteomyelitis 34. Notably, T‐MSCs constitutively secreted high levels of IL‐1Ra, but BM‐MSCs and AT‐MSCs exhibited a deficiency in IL‐1Ra production, which could enable the use of either T‐MSCs or T‐CM in the treatment of disease, considering the diverse paracrine effects of the cells alone or of the T‐CM after infusion. Considering that some MSC markers vary according to the tissue of origin 35, 36, we believe that IL‐1Ra may be a specific positive marker to distinguish T‐MSCs from MSCs derived from other tissues. Particularly, palatine tonsil is secondary lymphoid tissue that continuously encounters antigens and subsequently drive efficient immune response. Thus, we speculate those tissue specificity may render intrinsic priority to T‐MSCs in terms of immune regulatory properties. Our findings related to the interaction of muscle cells and fibroblasts under FFA‐rich conditions might expand the current therapeutic options for normalizing muscle architecture and function by furthering understanding of the pathophysiological processes involved in skeletal muscle tissue in obesity‐related diseases. As well, the impact of T‐CM on that process implied that T‐CM could facilitate tissue regeneration *via* its superior immunomodulatory function. We believe that T‐CM could also be beneficial to the treatment of inflammatory muscle diseases such as inflammatory myopathy, which is characterized by the development of fibrotic muscle tissue.

In summary, we demonstrated that T‐CM from human palatine T‐MSCs ameliorated the activation of fibroblasts induced by PA‐stimulated myotubes *via* the blockage of IL‐1β activity using the immune modulatory mediator, IL‐1Ra. These findings suggest that T‐CM might be a promising therapeutic for targeting inflammation associated with muscle inactivity.

## Conflicts of interest

The authors declare no conflicts of interests.

## Supporting information


**Figure S1.** T‐CM negatively regulate the expression of TLRs on myotubes. C2C12 myotubes on day 6 of 7 days of differentiation period were supplemented with T‐CM for 24 hrs. On the following day, the expression of TLR1‐9 was observed by RT‐PCR. TLR2, TLR3, TLR4 and TLR6 on myotubes treated with T‐CM were significantly decreased compared to those of non‐treated C2C12 myotubes. The data are presented as the mean ± SEM (*P < 0.05).

**Figure S2.** T‐CM contains approximately 100 ng of IL‐1Ra within 20 μl. 800 ng of rIL‐1Ra and 20 μl of T‐CM were loaded and the band pixel densities were calculated by using UN‐SCAN‐IT‐gel 6.1 software (Silk Scientific, Inc.). Recombinant IL‐1Ra showed eight times higher pixel densities than T‐CM.

**Figure S3.** Recombinant IL‐1Ra effectively inhibit the migration of L929 cells towards PA‐treated myotubes. To confirm dose dependent effect of rIL‐1Ra on L929 cells migration toward PA‐treated myotubes, myotubes were pre‐treated with 750 µM PA overnight in the bottom chamber and the media was changed after the addition of L929 cells in the upper chamber. Recombinant IL‐1Ra were added to the myotubes at a concentration of 0, 10, 25, 50, and 100 ng/ml. After 10 hrs, L929 cells that passed through the insert membrane and adhered to the opposite side were stained with crystal violet solution. Original magnification, 200×.
