# Supplementary figures and images for: Conditioned media from human palatine tonsil mesenchymal stem cells regulates the interaction between myotubes and fibroblasts by IL‐1Ra activity
Source: J Cell Mol Med. 2016 Sep 13;21(1):130–41. doi: 10.1111/jcmm.12947 (PMC5192826; doi:10.1111/jcmm.12947)

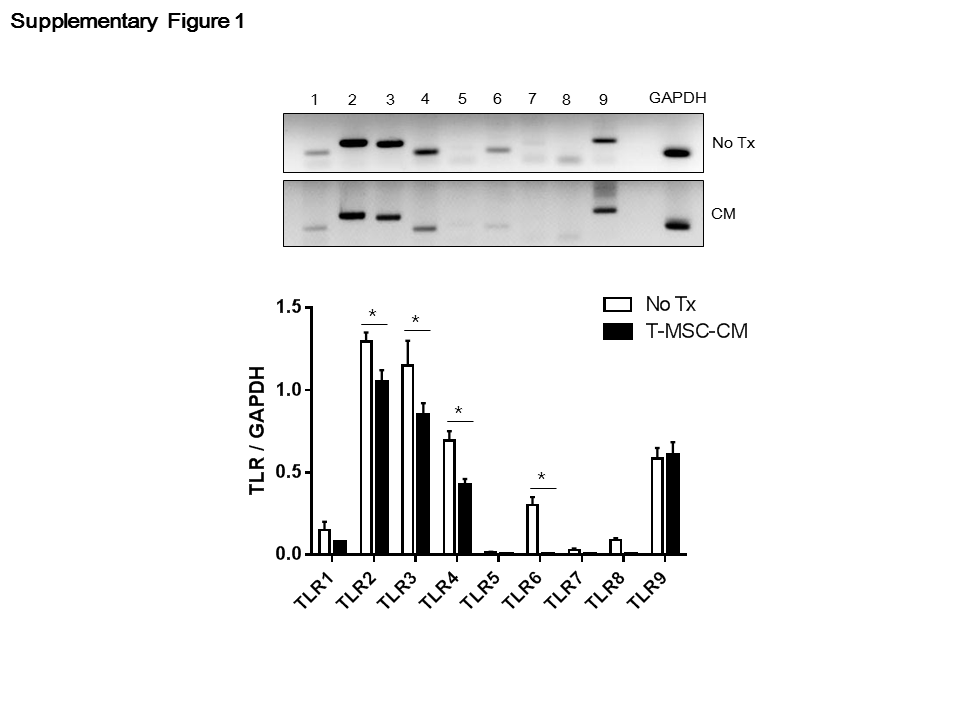

Supplement: Supplementary file 1 — Figure S1. T‐CM negatively regulate the expression of TLRs on myotubes. C2C12 myotubes on day 6 of 7 days of differentiation period were supplemented with T‐CM for 24 hrs. On the following day, the expression of TLR1‐9 was observed by RT‐PCR. TLR2, TLR3, TLR4 and TLR6 on myotubes treated with T‐CM were significantly decreased compared to those of non‐treated C2C12 myotubes. The data are presented as the mean ± SEM (*P < 0.05). [file JCMM-21-130-s001.tif]

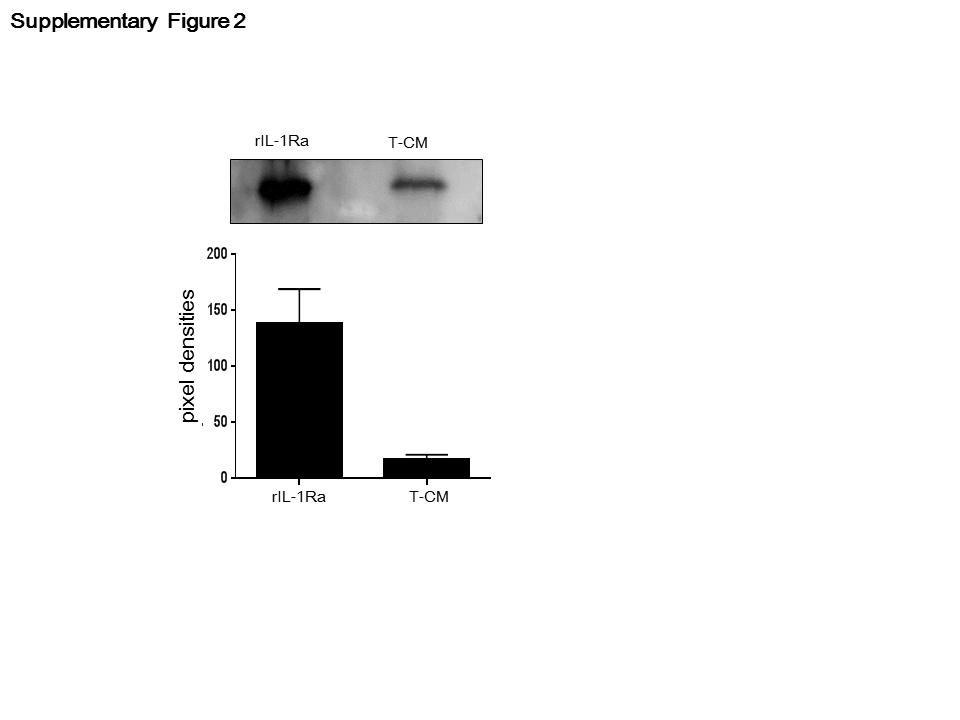

Supplement: Supplementary file 2 — Figure S2. T‐CM contains approximately 100 ng of IL‐1Ra within 20 μl. 800 ng of rIL‐1Ra and 20 μl of T‐CM were loaded and the band pixel densities were calculated by using UN‐SCAN‐IT‐gel 6.1 software (Silk Scientific, Inc.). Recombinant IL‐1Ra showed eight times higher pixel densities than T‐CM. [file JCMM-21-130-s002.tif]

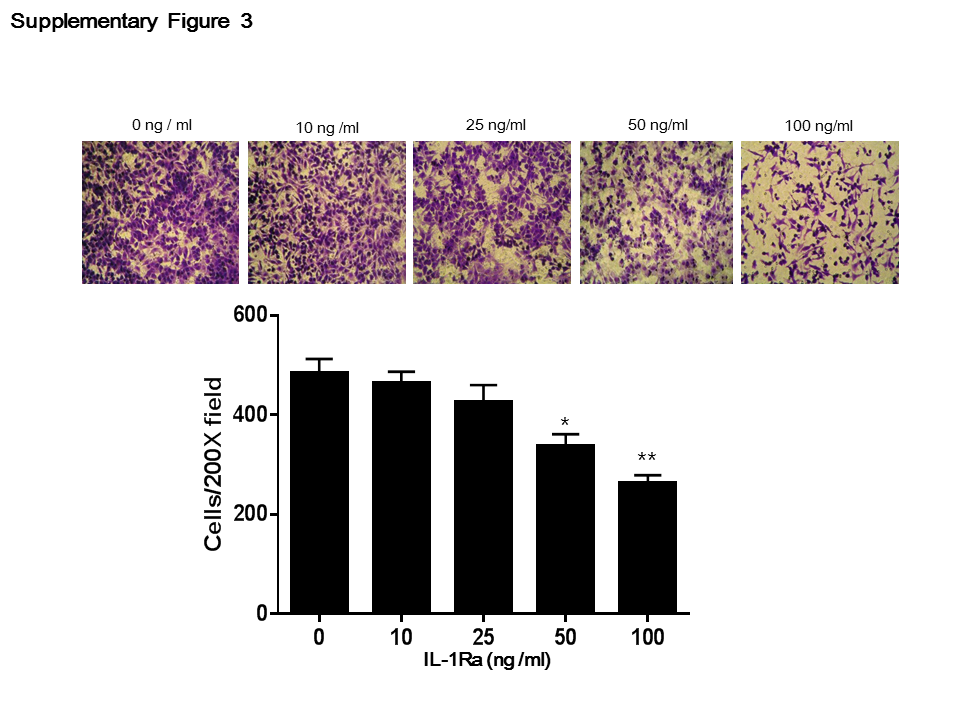

Supplement: Supplementary file 3 — Figure S3. Recombinant IL‐1Ra effectively inhibit the migration of L929 cells towards PA‐treated myotubes. To confirm dose dependent effect of rIL‐1Ra on L929 cells migration toward PA‐treated myotubes, myotubes were pre‐treated with 750 µM PA overnight in the bottom chamber and the media was changed after the addition of L929 cells in the upper chamber. Recombinant IL‐1Ra were added to the myotubes at a concentration of 0, 10, 25, 50, and 100 ng/ml. After 10 hrs, L929 cells that passed through the insert membrane and adhered to the opposite side were stained with crystal violet solution. Original magnification, 200×. [file JCMM-21-130-s003.tif]
